# Dysregulated miR-21/SOD3, but Not miR-30b/CAT, Profile in Elderly Patients with Carbohydrate Metabolism Disorders: A Link to Oxidative Stress and Metabolic Dysfunction

**DOI:** 10.3390/ijms26094127

**Published:** 2025-04-26

**Authors:** Adam Włodarski, Izabela Szymczak-Pajor, Jacek Kasznicki, Egle Morta Antanaviciute, Bożena Szymańska, Agnieszka Śliwińska

**Affiliations:** 1 Department of Nucleic Acid Biochemistry, Medical University of Lodz, 92-213 Lodz, Poland; adam.wlodarski@stud.umed.lodz.pl (A.W.); izabela.szymczak@umed.lodz.pl (I.S.-P.); 2 Department of Internal Diseases, Diabetology and Clinical Pharmacology, Medical University of Lodz, 92-213 Lodz, Poland; jacek.kasznicki@umed.lodz.pl; 3 Centre for Cellular Microenvironments, Mazumdar-Shaw Advanced Research Centre, University of Glasgow, Glasgow G12 8QQ, UK; 4 CoreLab, Central Scientific Laboratory of the Medical University of Lodz, Mazowiecka 6/8 St., 92-215 Lodz, Poland; bozena.szymanska@umed.lodz.pl

**Keywords:** SOD3, CAT, miR-21, miR-30b, microRNA, oxidative stress, T2DM, prediabetes, carbohydrate metabolism disorders, aging

## Abstract

Carbohydrate metabolism disorders (CMDs), including prediabetes and type 2 diabetes mellitus (T2DM), are increasingly prevalent in the aging population. Oxidative stress (OxS) plays a pivotal role in CMD pathogenesis, with extracellular superoxide dismutase (SOD3) and catalase (CAT) serving as critical antioxidant defenses. Additionally, microRNAs (miR-21 and miR-30b) regulate the oxidative and inflammatory pathways, yet their roles in elderly CMD patients remain unclear. This study evaluated miR-21 and miR-30b expression alongside SOD3 and CAT plasma levels in individuals aged ≥ 65 years (n = 126) categorized into control (n = 38), prediabetes (n = 37), and T2DM (n = 51) groups. Quantitative PCR assessed miRNA expression, while ELISA measured the enzyme levels. SOD3 levels were significantly reduced in CMDs, particularly in T2DM, whereas miR-21 was upregulated. A negative correlation between SOD3 and miR-21 was strongest in T2DM, suggesting a regulatory interplay. Neither CAT levels nor miR-30b expression differed among groups. Logistic regression indicated SOD3 as a protective biomarker, with each 1 ng/mL increase reducing the CMD risk by ~5–6%. The ROC analysis supported SOD3’s diagnostic potential, while miR-21 showed a modest association. These findings highlight SOD3 downregulation and miR-21 upregulation as potential contributors to CMD progression in elderly patients, warranting further research into their mechanistic roles and therapeutic potential.

## 1. Introduction

Carbohydrate metabolism disorders (CMDs), including prediabetes and type 2 diabetes mellitus (T2DM), are a growing global health challenge due to their asymptomatic onset and the risk of severe complications [1,2]. Traditional diagnostic tools, including fasting plasma glucose (FPG), glycated hemoglobin (HbA1c), and glucose tolerance tests, primarily detect CMDs at advanced stages, underscoring the need for more sensitive biomarkers for early detection [2].

Oxidative stress (OxS), characterized by an imbalance between reactive oxygen species (ROS) production and antioxidant defense mechanisms, plays a pivotal role in the pathogenesis of CMDs [3]. In the setting of chronic hyperglycemia (HG), increased mitochondrial ROS generation, intensified activity of the polyol pathway, and the subsequent formation of advanced glycation end products (AGEs) collectively damage cellular proteins, lipids, and DNA, promoting inflammatory processes and accelerating senescence [4,5]. These DNA damages, including double-strand breaks, lead to the activation of a robust DNA damage response signaling through the pathways of p53/p21 and p16INK4a [6]. The activation of these pathways puts cells into senescence, a permanent loss of the ability to divide, which impairs tissue regeneration [6]. Furthermore, chronic HG also promotes the glycation of proteins and lipids, producing AGEs that bind RAGE receptors and trigger nuclear factor κB (NF-κB)-mediated chronic inflammation [7,8]. Additionally, AGEs downregulate the endogenous antioxidant systems, including superoxide dismutase (SOD), catalase (CAT), and glutathione peroxidase (GPx), making cells more exposed to OxS [9]. This vulnerability exacerbates extracellular matrix degradation and weakens tissue barrier functions in structures such as the endothelium, pancreas, and visceral adipose tissue [9,10,11]. Chronic HG triggers OxS, which further exacerbates HG, creating a self-perpetuating vicious cycle that accelerates additional systemic aging.

Antioxidant enzymes serve as the front-line defense against ROS. The SOD family, comprising SOD1 or CuZn-SOD (cytosolic), SOD2 or Mn-SOD (mitochondrial), and SOD3 or EC-SOD (extracellular), catalyzes the conversion of the superoxide radical (O_2_^−^) to hydrogen peroxide (H_2_O_2_) [12], which is subsequently degraded by CAT located primarily in peroxisomes. Among these, SOD3 is of particular interest due to its extracellular localization. Additionally, SOD3 preserves the bioactivity of nitric oxide and facilitates hypoxia-induced gene expression in the visceral adipose tissue [13].

CAT is a heme-containing enzyme critical for mitigating the cytotoxic effects of hydrogen peroxide accumulation [14,15]. This damage disrupts insulin signaling, initially promoting insulin resistance (IR) and ultimately leading to beta-cell dysfunction and failure [16,17,18]. As we partially demonstrated previously, altered levels of these antioxidant enzymes, including GPx3, are implicated in the progression of CMDs and may serve as potential biomarkers for disease monitoring [19].

In the past decade, numerous studies have highlighted a potential relationship between OxS, HG, and microRNAs (miRNAs)—small non-coding RNAs that regulate gene expression by binding to the 3′ untranslated regions (3′UTRs) of target mRNAs, leading to translational repression or mRNA degradation [20,21,22,23,24]. Notably, the expression of these miRNAs is often altered in key metabolic organs—such as the pancreas, white adipose tissue, and skeletal muscle—which can interfere with the maintenance of metabolic balance [20,23]. Moreover, their stability and consistent expression patterns among individuals of the same ethnic group make miRNAs promising biomarkers for T2DM, offering potential advancements in disease monitoring [23].

Chronic HG and OxS alter miRNA biogenesis and stability through redox-sensitive transcription factors (e.g., NF-κB, AP-1, p53), as well as via epigenetic and post-transcriptional modifications [20,23]. Moreover, they can impair the functions of proteins like Drosha, Dicer, and Argonaute, disrupting proper miRNA maturation and the loading of miRNAs into the RNA-induced silencing complex (RISC), disrupting their regulatory roles in loading [21,25,26].

miR-21, located on chromosome 17q23.2, is one of the most studied microRNAs, the overexpression of which promotes tumorigenesis and metabolic disorders in both human cells and mouse models [27]. miR-21 significantly contributes to T2DM by modulating key pathways that worsen insulin resistance and pancreatic beta-cell dysfunction by modulating pathways such as EGFR/Akt, Ras/MAPK (via Sprouty 2), and PI3K/Akt/NF-κB [27,28,29,30]. A critical mechanism involves directly inhibiting SOD3 by binding to the 3′UTR of SOD3 mRNA [31], resulting in diminished antioxidant defense, increased levels of ROS, and triggering a feedback loop that elevates miR-21 expression.

Although miR-30b is less extensively studied than some other microRNAs in T2DM, emerging evidence indicates that its upregulation in adipose tissue contributes to dysregulated adipogenesis and IR [32,33]. Notably, Haque et al. demonstrated that miR-30b binds to the 3′-UTR of human catalase mRNA [34]. Drawing upon prior findings and an in-depth literature review—supported by bioinformatic predictions and dual-luciferase reporter assay data—we determined that miR-21 and miR-30b act as suppressors of SOD3 and CAT expression [31,34].

In this study, we examined the relative expression of miR-21 and miR-30b alongside serum SOD3 and CAT levels in elderly (65 years and older) individuals with CMDs. By correlating these molecular markers with anthropometric (BMI, WHR, and bioimpedance) and metabolic (renal, glycemic, and lipid) parameters, we aim to clarify how microRNA dysregulation and diminished antioxidant defense contribute to metabolic dysfunction. Our focus on this population addresses their elevated risk, weakened antioxidant systems, and the paucity of literature data, ultimately guiding earlier detection and better management of CMDs.

## 2. Results

### 2.1. Characteristics of the Participants

Briefly, adult participants aged 65 years and older were stratified into controls (n = 38), prediabetes (n = 37), and T2DM (n = 51) based on the American Diabetes Association (ADA 2019 [35]) diagnostic criteria. Detailed demographic, anthropometric, and metabolic characteristics can be found in Table 1 and in Table S1 in Supplementary Materials.

The study groups showed no significant differences in age, sex, height, systolic blood pressure (SBP), diastolic blood pressure (DBP), and various anthropometric parameters, including body mass index (BMI), waist–hip ratio (WHR), and visceral fat rating. As expected, patients with T2DM had significantly higher measurements in all three skinfold thicknesses assessed (triceps, abdominal, and thigh). Moreover, T2DM patients showed a thicker triceps skinfold compared to those with prediabetes and control.

In our assessment of carbohydrate metabolism, both the prediabetes and T2DM groups exhibited significantly elevated levels of fasting plasma glucose (FPG) and glycated hemoglobin (HbA1c) compared to the control group. Notably, FPG was markedly higher in the T2DM group than in the prediabetes group, while HbA1c levels in the latter remained significantly above those observed in controls. An evaluation of insulin resistance revealed a pronounced increase in HOMA-IR values among individuals with T2DM relative to the control group. With regard to the lipid profiles, the T2DM group demonstrated a significant reduction in high-density lipoprotein (HDL) cholesterol levels compared to controls; however, no substantial intergroup differences were observed for other lipid parameters. The observed decreases in low-density lipoprotein (LDL) cholesterol and total cholesterol (TC) levels in the T2DM group may reflect the use of statins, particularly inhibitors of hydroxymethylglutaryl-CoA (HMG-CoA) reductase. Renal function analysis indicated significantly elevated urea concentrations in the T2DM group versus controls, while other renal markers—including serum creatinine and estimated glomerular filtration rate (eGFR)—remained stable across all groups.

Bioelectrical impedance analysis (BIA) is a widely used, non-invasive, objective, and cost-effective body composition assessment method with high reproducibility [36]. The BIA method enables precise determination of body fat percentage, fat-free mass, and hydration levels. In clinical and population studies, BIA demonstrates high reproducibility and accuracy, making it a valuable tool for monitoring changes in body composition under various health conditions. As anticipated, participants with T2DM demonstrated a significantly higher percentage of body fat compared to both the control and prediabetes groups. Additionally, the T2DM group showed markedly lower proportions of muscle tissue and total body water relative to controls. However, no statistically significant differences were observed among the groups in the absolute (kilogram) values of body fat, muscle mass, or total body water.

The oral antihyperglycemic and lipid-lowering medications administered to the study participants have been detailed in our previous publication and in Table S2 in Supplementary Materials [19]. In summary, within the control group, half of the individuals were prescribed statins. These medications are primarily used to manage hypercholesterolemia by decreasing the levels of total TC, LDL, and triglycerides (TG) while increasing HDL concentrations. In the prediabetes group, more than 50% of the participants were also receiving statin therapy. Dietary management was the sole therapeutic approach for nearly 40% of the individuals with prediabetes, with metformin prescribed to only two participants. In contrast, metformin was the primary treatment in the T2DM group. About one-third of the T2DM patients received dual therapy involving antidiabetic agents and/or statins, while another third were on polypharmacy regimens comprising three or more medications.

### 2.2. Relative Expression of miR-21 and SOD3 Levels

**Figure 1:**
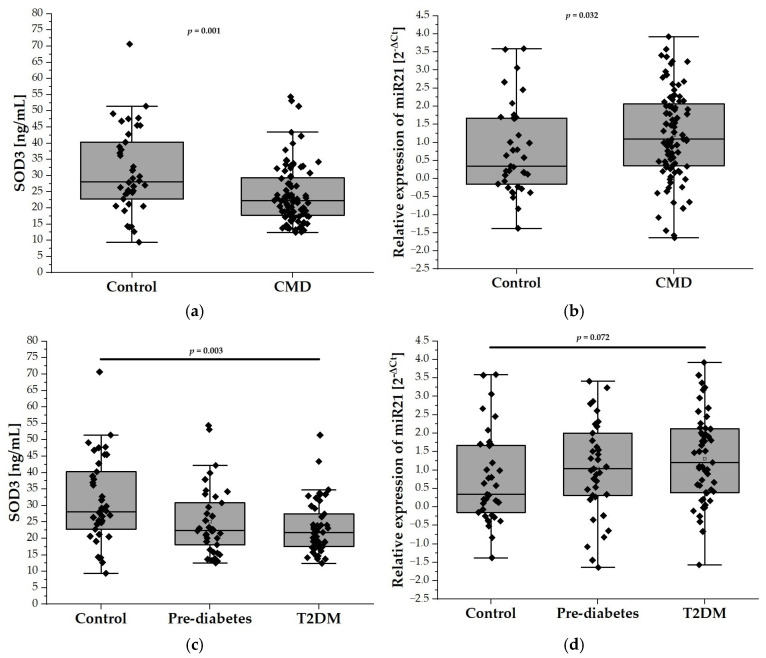
The plasma levels of SOD3 and the relative expression of miR-21 in our study population (n = 126). Panel (**a**) compares the plasma levels of SOD3 in the control group (n = 38) with the CMD group (n = 88), while panel (**c**) illustrates the levels among control (n = 38), prediabetes (n = 37), and T2DM (n = 51) participants aged 65 and older. Panel (**b**) compares relative expression of miR-21 in the control group (n = 38) with the CMD group ( n = 88), while panel (**d**) compares relative expression of miR-21 in the control (n = 38), prediabetes (n = 37), and T2DM (n = 51) groups. Data are presented as medians with interquartile ranges and minimum and maximum values, depicted through box-and-whisker plots overlaid with dot plots.

In our analysis, the plasma levels of SOD3 were found to be significantly lower in the CMDs group compared to the control group (31.33 ± 13.13 ng/mL vs. 24.04 ± 9.04 ng/mL; *p* < 0.001), as depicted in Figure 1a. Further examination revealed that SOD3 levels were reduced in both the prediabetes and T2DM groups when compared to controls. Notably, a statistically significant difference was observed between the T2DM patients and the control group (31.33 ± 13.13 ng/mL vs. 23.21 ± 7.85 ng/mL; *p* < 0.01), suggesting a marked reduction of SOD3 levels in the presence of T2DM (Figure 1c). Notably, the relative expression level of miR-21 was significantly higher in the CMDs group compared to the control group (0.7763 ± 0.5342 vs. 0.6212 ± 0.5972; *p* < 0.05) (Figure 1b). Furthermore, although the relative expression of miR-21 was higher in the prediabetes and T2DM groups compared to the control group, these differences did not reach statistical significance (Figure 1d).

### 2.3. Relative Expression of miR-30b and CAT Levels

**Figure 2:**
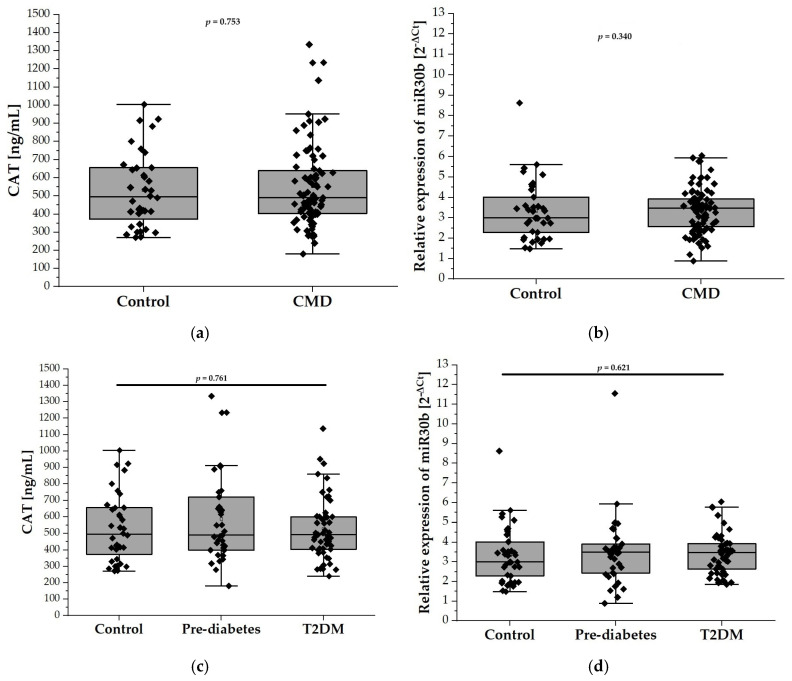
The plasma levels of CAT and relative expression of miR-30b in our study population (n = 126). Panel (**a**) compares the plasma levels of CAT in the control group (n = 38) with the CMD group (n = 88), while panel (**c**) illustrates the levels among control (n = 38), prediabetes (n = 37), and T2DM (n = 51) participants aged 65 and older. Panel (**b**) compares relative expression of miR-30b in the control group (n = 38) with the CMD group (n = 88), while panel (**d**) compares relative expression of miR-30b in the control (n = 38), prediabetes (n = 37), and T2DM (n = 51) groups. Data are presented as medians with interquartile ranges and minimum and maximum values, depicted through box-and-whisker plots overlaid with dot plots.

The plasma levels of CAT, measured by ELISA, did not differ significantly between the control group and patients with CMDs (532.5 ± 204.9 U/mL vs. 553.3 ± 229.4 U/mL; Figure 2a). Similarly, stratification of the CMD group into prediabetes and T2DM subgroups did not reveal any differences in the plasma CAT levels between these subgroups. (Figure 2c). Figure 2b,d depict the relative expression levels of miR-30b among the study groups. Specifically, Figure 2b compares the control group with individuals diagnosed with CMDs, while Figure 2d illustrates the expression levels in the prediabetes and T2DM groups. Statistical analysis revealed no significant differences in miR-30b expression between the control group and the CMDs group, nor between the control group and the prediabetes and T2DM groups.

### 2.4. Correlation Between SOD3 and miR-21 Levels and Anthropometric and Metabolic Parameters

Table S3a presents Spearman’s correlations between SOD3 levels and miR-21 expression, SBP, DBP, and various anthropometric parameters. In the control group, no significant correlation was observed between the SOD3 levels and miR-21 expression. Conversely, in the CMDs group, a weak negative correlation was identified between the SOD3 levels and miR-21 expression, which intensified to a moderate negative correlation in the T2DM subgroup. In the control group, SOD3 levels exhibited a moderate negative correlation with age and positive correlations with body height and total body water percentage. In contrast, the CMDs group showed a weak negative correlation between SOD3 levels and age and positive correlations with both SBP and DBP. Further analysis of the prediabetes and T2DM subgroups revealed a moderate negative correlation between SOD3 levels and age in the prediabetes group, a weak positive correlation between SOD3 levels and SBP in the prediabetes group, and a moderate negative correlation between SOD3 levels and total body water percentage in the T2DM group.

As detailed in Table S3b, in the control group, no significant correlations were observed between SOD3 levels and metabolic parameters, despite the fact that SOD3 levels demonstrated moderate negative correlations with creatinine and urea concentrations and a moderate positive correlation with eGFR. In the CMDs group, no significant correlations were found between SOD3 levels and the metabolic or renal parameters. In the subgroup analysis of patients with prediabetes and T2DM, a moderate negative correlation between SOD3 levels and HDL concentration was observed only in the prediabetes group.

In Table S3c, we analyzed correlations between miR-21 expression and various anthropometric parameters. In the control group, miR-21 expression was significantly negatively correlated with age and BMI. It also showed inverse relationships with adiposity measures, such as ST at the triceps, abdominal region, and thigh, as well as with visceral fat rating and BIA-BF%. Conversely, there was a positive correlation between miR-21 expression and BIA-TBW% and BIA-FFM%. In the CMDs group, significant negative correlations were found between miR-21 expression and ST at the triceps and thigh, as well as BIA-BF%. Positive correlations emerged with BIA-FFM%, BIA-TBW%, and BIA-TBW (kg). In the prediabetes subgroup, miR-21 expression was positively correlated with height, BIA-FFM%, BIA-FFM (kg), and total body water in kilograms. In the T2DM group, a significant negative correlation was observed only with ST at the triceps.

Table S3d presents the correlations between miR-21 expression levels and various metabolic and renal parameters. The statistical analyses revealed no significant correlations between miR-21 expression and any of these parameters within any of the groups studied.

Overall, these findings suggest that SOD3 and miR-21 may play complementary roles in the pathophysiology of CMDs in elderly individuals. In particular, the stronger negative correlation between SOD3 and miR-21 in the T2DM subgroup points to a more pronounced interplay between oxidative stress pathways (SOD3) and post-transcriptional regulation (miR-21) in the advanced stages of dysglycemia. By underlining the negative correlations of both SOD3 and miR-21 with age and emphasizing their indirect regulatory link, these findings provide a strong rationale for exploring miR-21–SOD3 modulation as a target for intervention in elderly patients with CMDs.

### 2.5. Correlation Between CAT and miR30b Levels and Anthropometric and Metabolic Parameters

Table S4a presents the correlations between anthropometric measurements, SBP, and DBP and the expression levels of miR-30b and CAT levels. No statistically significant associations were observed between miR-30b expression and CAT levels across the study groups.

In the control group, significant negative correlations were observed between CAT and SBP, DBP, ST triceps, ST thigh, and BIA-BF [%]. Positive correlations were noted with measures of lean mass and hydration status. In the CMDs and prediabetes groups, CAT levels showed significant inverse correlations with body mass. No significant associations were found in the T2DM group.

An analysis of the correlations between CAT levels and the metabolic as well as renal parameters is provided in Table S4b. None of the assessed parameters of carbohydrate and lipid metabolism in the studied groups correlated significantly with the plasma CAT levels. In the control group, CAT levels showed a strong negative correlation with creatinine and a moderate positive correlation with eGFR. In the CMDs group, CAT levels exhibited a weak negative correlation with creatinine and urea, which became more pronounced in the T2DM subgroup. Additionally, in the T2DM subgroup, CAT levels demonstrated a moderate positive correlation with eGFR.

No statistically significant correlations between miR-30b expression levels and the anthropometric or metabolic parameters were observed in the studied groups, as presented in Table S4c,d.

### 2.6. Diagnostic Utility of Plasma SOD3 Concentration and miR-21 Expression in Assessing CMD Risk

In our analysis, SOD3 emerged as a significant predictor of CMD risk (Table 2). After excluding outliers, each 1 ng/mL increase in SOD3 was associated with approximately a 6% reduction in CMD odds in the univariate model (OR = 0.941; 95% CI: 0.906–0.976; *p* = 0.001) and a 5% reduction in the multivariate model (OR = 0.948; 95% CI: 0.909–0.988; *p* = 0.012). The results suggest that SOD3 may serve as a protective biomarker in aging individuals at risk of metabolic dysfunction.

Among other analyzed parameters, the univariate analysis showed the increased risk of CMD for higher values of ST triceps (OR = 1.062; 95% CI: 1.010–1.116; *p* = 0.018), ST abdominal (OR = 1.034; 95% CI: 1.011–1.069; *p* = 0.043), and HOMA-IR (OR = 1.794; 95% CI: 1.012–3.179; *p* = 0.045). However, these associations became non-significant in the multivariate model. As we expected, FPG exhibited a strong, positive association with CMD, showing high significance in both univariate (OR = 2.585; CI = 1.500–4.456; *p* < 0.001) and multivariate models (OR = 2.915; CI = 1.484–5.726; *p* = 0.002), indicating increased odds of CMD with elevated FPG levels.

**Figure 3:**
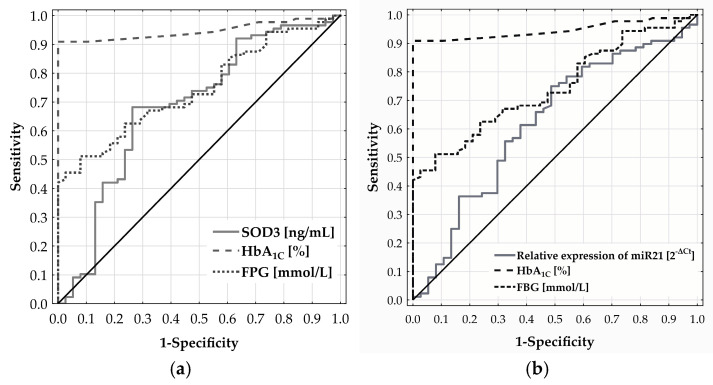
Diagnostic performance of SOD3 (**a**) and miR-21 (**b**) in predicting CMD risk in adults aged 65 and older, as assessed by ROC curve analysis.

ROC analysis was used to assess the ability of SOD3 and miR-21 levels to discriminate CMD risk in adults aged 65 and above. For SOD3, the ROC curve yielded an AUC of 0.684 (*p* = 0.0008) (Figure 3a) with the optimal cut-off at 24.132 ng/mL, determined by Youden’s index. Patients with SOD3 levels below this threshold had a significantly higher CMD risk—over 5.7 times that of patients with higher levels (OR = 5.697; 95% CI: 2.440–13.300; *p* < 0.0001). Similarly, the ROC curve for miR-21 expression produced an AUC of 0.622, reflecting a modest discriminatory ability (Figure 3b). The optimal cut-off at 0.768 [2^−ΔCt^] was associated with a more than three-fold increase in CMD risk (OR = 2.983; 95% CI: 1.339–6.646; *p* = 0.0118).

### 2.7. Evaluation of Plasma CAT Levels and miR-30b Expression as Predictive Biomarkers of CMD in the Elderly

Neither plasma CAT nor miR-30b expression showed significant diagnostic relevance for CMD in individuals aged 65 and above, with ROC analysis yielding AUCs of 0.518 (*p* = 0.751) and 0.552 (*p* = 0.358), respectively.

## 3. Discussion

Our study focused on the expression of two microRNAs (miR-21 and miR-30b) and the plasma levels of their respective mRNA targets encoding the antioxidant enzymes SOD3 and CAT in older adults (>65 years) with varying degrees of CMDs. The main observations highlight (i) a significant decrease in SOD3 in CMDs, most pronounced in T2DM, (ii) a notable elevation of miR-21 in the overall CMD group versus controls, (iii) no substantial changes in CAT levels or miR-30b expression across the study groups, and (iv) negative correlations between SOD3 and miR-21 that became stronger in T2DM. Additionally, logistic regression and ROC curve analyses point to SOD3 (and, to a lesser degree, miR-21) as potential biomarkers of CMD risk in older adults, whereas CAT and miR-30b showed limited diagnostic utility.

CMDs are increasingly prevalent, primarily driven by age-related metabolic decline and the global obesity epidemic. Despite the availability of diagnostic indices such as FPG, HbA1c, glucose tolerance tests, and HOMA-IR, early-stage CMD often escapes clinical detection due to its insidious onset. This latency in diagnosis permits disease progression and substantially increases the risk of vascular complications in unrecognized cases [37].

Advanced age is frequently associated with a gradual decline in the efficiency of endogenous antioxidant systems, including SOD, CAT, and GPx, all of which help maintain redox homeostasis [38,39]. Chronic hyperglycemia (as seen in prediabetes and T2DM) further amplifies this vulnerability by generating excessive ROS through multiple pathways, such as NADPH oxidase or mitochondrial electron transport chain overload [4]. The extracellular isoform SOD3 is particularly important in older individuals because it preserves nitric oxide (NO) bioavailability and limits oxidative damage in the extracellular matrix, thereby preventing peroxynitrite formation and endothelial and mitochondrial dysfunction [40]. Our findings emphasize a pronounced decrease in SOD3 in participants with T2DM, aligning with previous reports showing that impairments in antioxidant capacity are exacerbated by hyperglycemia [41,42]. Interestingly, Lewandowski et al. [43] observed higher SOD3 levels in patients with T2DM compared to their non-diabetic controls. However, possible differences in participant age ranges, T2DM duration, single nucleotide polymorphisms (SNPs), and medication use may further contribute to the conflicting SOD3 findings between the two studies. Genetic factors, including SNPs in the genes encoding SOD isozymes, can influence individual variations in SOD levels and activity. For instance, the rs1799895 variant in SOD3 involves an Arg213Gly substitution that reduces its affinity for heparan sulfate in the extracellular matrix, thereby increasing its concentration in the plasma [44].

In this study, miR-21 expression was significantly higher in the CMDs group than in controls, with an upward trend evident in both prediabetes and T2DM subgroups. Although sample size constraints may have limited the statistical power of subgroup analyses, miR-21 still correlated with body composition indicators (e.g., BMI, lean mass) but showed no direct associations with core metabolic or renal parameters. This observation suggests that miR-21 alone may offer limited value as an independent marker of CMD, even though prior reports broadly support the upregulation of miR-21 in T2DM [45,46,47,48], with a few studies indicating a reduced miR-21 in diabetes [49]. Recent data highlight Krev/Rap1 interaction trapped-1 (KRIT1) as a key defensive factor in endothelial homeostasis by engaging the ERK–NRF2 pathway, ensuring proper antioxidant responses [50]. La Sala et al. demonstrated that under hyperglycemic conditions, miR-21 upregulates and suppresses KRIT1, thus diminishing ERK-driven NRF2 activation and lowering SOD2 expression—a mechanism that can further amplify miR-21’s inhibition of SOD3 [47]. Consequently, targeting the miR-21–KRIT1–ERK–NRF2 axis could bolster antioxidant defenses, curb oxidative damage, and help mitigate diabetic complications. Mechanistically, experimental evidence indicates that miR-21 binds the 3′UTR of SOD3 mRNA, thereby inhibiting SOD3 translation and weakening antioxidant defenses [31]. Meanwhile, ROS generated under chronic inflammation can further promote miR-21 expression: specifically, NF-κB (nuclear factor kappa B) and AP-1 (activator protein-1), both redox-sensitive transcription factors, bind to regulatory regions of the miR-21 gene, effectively amplifying or stabilizing its transcription [31,51,52]. Our findings reveal a stronger negative correlation between miR-21 and SOD3 in T2DM, underscoring how “inflammaging,” hyperglycemia, and miR-21–SOD3 dysregulation jointly escalate OxS. Consequently, targeting this miR-21–SOD3 axis may offer a promising strategy to alleviate oxidative damage and improve metabolic outcomes in vulnerable populations.

From a clinical standpoint, our ROC analyses showed that each 1 ng/mL increase in SOD3 significantly reduces the CMD odds by 5–6% in older adults, while miR-21 had a modest but noteworthy capability to distinguish CMD patients from controls. Given that age and suboptimal glycemic control often coexist in driving oxidative stress, measuring SOD3 (and possibly miR-21) in tandem with established tests like FPG or HbA1c could improve the early detection of dysglycemia. These biomarkers may capture oxidative and proinflammatory shifts before classical glucose thresholds are reached, ultimately enhancing risk stratification and targeting interventions like dietary changes, exercise programs, or antioxidant therapies in geriatric populations.

Interestingly, in our control cohort, we observed a negative correlation between miR-21 and age, BMI, all measured skinfolds (triceps, abdomen, thigh), visceral fat rating, and BIA-derived body fat percentage, alongside a positive correlation with BIA-FFM and BIA-TBW. However, most of these associations weakened or disappeared in the CMD group, leaving only a single negative correlation (triceps skinfold) in T2DM. Notably, our findings align with the report by Ghorbani et al., who also demonstrated an inverse correlation between miR-21 and BMI [53]. In contrast, many in vitro and animal studies show that miR-21 levels increase with rising BMI [54,55], suggesting that differences in study models, metabolic states, and tissue-specific expression may lead to seemingly opposite outcomes.

Unlike SOD3, the plasma levels of CAT did not differ significantly between the control and CMD groups in this cohort, and we also did not observe robust correlations between miR-30b and CAT or other metabolic parameters. While CAT is essential for detoxifying hydrogen peroxide generated by SOD-mediated reactions [9], several compensatory antioxidant pathways (e.g., GPxs, peroxiredoxins) might mask any CAT deficits in circulation. Moreover, miR-30b may exert its effects more prominently within the adipose or muscle tissues rather than in plasma, a possibility supported by literature highlighting its role in adipogenesis and insulin resistance [32,33].These findings indicate that CAT and miR-30b are less likely to serve as systemic biomarkers for CMDs in older individuals.

Despite the robustness of our findings, several limitations should be acknowledged. The cross-sectional design limits causal inferences, requiring prospective studies to determine whether declining SOD3 and elevated miR-21 are drivers or mere byproducts of CMDs. Additionally, the limited control group size and the lack of gender adjustment due to small subgroup numbers may impact the generalizability of results. Medication use, such as statins and metformin, could confound the observed associations, warranting larger cohorts to clarify these effects. The absence of direct SOD3 and CAT gene expression measurements, constrained by financial limitations, also represents a methodological gap.

The key strengths of this study include the following: (1) the use of human samples obtained from an elderly population, a group that is rarely examined in miRNA research, and (2) the comprehensive collection of anthropometric and metabolic data, which serves as a valuable foundation for future investigations and meta-analyses. Nonetheless, further clinical studies with a larger sample size are necessary to enhance the robustness of these findings.

## 4. Materials and Methods

This study enrolled 126 participants from an initially screened cohort of 131 individuals. Recruitment took place between January 2019 and February 2022 at the Department of Internal Medicine, Diabetology, and Clinical Pharmacology, Medical University of Lodz. The study was approved by the Bioethics Committee of the Medical University of Lodz (approval no. RNN/193/18/KE; 18 May 2018) and conducted in accordance with the Declaration of Helsinki and Good Clinical Practice guidelines. All participants provided written informed consent prior to inclusion.

Eligible participants were required to be aged 65 years or older. Assignment to the CMD group was based on either a confirmed diagnosis of prediabetes or T2DM, as defined by the American Diabetes Association (ADA, 2019), or laboratory findings consistent with HbA1c ≥ 5.7% or fasting plasma glucose (FPG) > 5.5 mmol/L [35]. Participants of the same age range with HbA1c < 5.7% and FPG ≤ 5.5 mmol/L were included in the control group.

Exclusion criteria included individuals younger than 65, those with diabetes other than T2DM, and individuals with active or historical malignancies, recent radiotherapy or chemotherapy, or acute conditions marked by elevated CRP or leukocytosis. Additionally, participants with acute coronary syndrome, acute abdominal issues, exacerbations of COPD or asthma, active smoking status, a genetic predisposition or family history of genetic disorders, thyroid dysfunction, severe hepatic disease, or recent blood transfusions were excluded. Moreover, participants did not engage in physical activity prior to sample collection and do not have severe diabetes complications. Following this, participants’ medical histories were reviewed, and a thorough physical examination was conducted. Finally, five of the patients initially included in the study were excluded: two due to malignancy detection and three due to sepsis.

Eligible participants were stratified into two primary groups: CMD group (n = 88) and an age-matched control group (n = 38). The CMD group included individuals with prediabetes (n = 37) and T2DM (n = 51). Prediabetes was defined by FPG levels between 5.6 and 6.9 mmol/L or HbA1c values of 5.7–6.4%. The T2DM subgroup included participants with a confirmed clinical diagnosis, current treatment with hypoglycemic agents, FPG > 7.0 mmol/L, or HbA1c ≥ 6.5%.

Clinical parameters included SBP, DBP, body weight, height, WC, HC, BMI, and WHR. Fasting venous blood samples were analyzed for FPG, insulin, HbA1c, TC, LDL-C, HDL-C, TG, uric acid, and creatinine. eGFR was calculated using the MDRD equation. HbA1c was quantified by high-performance liquid chromatography and FPG by hexokinase-catalyzed photometric assay. Lipid panels (TC, HDL-C, TG) were measured using enzymatic colorimetric methods on Beckman Coulter AU analyzers (Beckman Coulter, Brea, CA, USA). LDL-C was derived via the Friedewald formula. Insulin was assessed by electrochemiluminescence immunoassay.

Collected samples of the blood were processed for the analysis of microRNA expression and measurements of SOD3 and CAT. After centrifugation at 3000× *g* for 10 min at 4 °C, plasma was aliquoted with QIAzol^®^ lysis reagent (Qiagen, Germantown, MD, USA) and stored at −80 °C for later RNA isolation using the miRNeasy Serum/Plasma Kit (Qiagen). The remaining plasma for SOD3 and CAT analyses was divided into 100 µL aliquots and stored at −80 °C for ELISA testing.

Anthropometric data were collected between 8:00 and 10:00 a.m. after an overnight fast, including weight, height, WC, and HC. BMI and WHR were calculated, and skinfold thickness was measured at three sites (triceps, umbilical region, quadriceps) using BATY^®^ calipers (BATY CE 0120, Baty International, Sheffield, South Yorkshire, UK). Bioelectrical impedance analysis (BIA) was performed with a TANITA MC-780MA scale (TANITA Corporation, Tokyo, Japan), measuring parameters like body fat, muscle mass, and total body water with precision.

### 4.1. Measurement of SOD3 and CAT Levels—ELISA

Plasma levels of SOD3 and CAT were measured and quantified using enzyme-linked immunosorbent assay (ELISA) kits (Wuhan Fine Biotech, Wuhan, Hubei, China) according to the manufacturer’s manuals. Serum samples were diluted 12-fold for CAT and 3-fold for SOD3 measurements.

### 4.2. Analysis of microRNA Expression—Quantitative Real-Time PCR Assay (qRT-PCR)

The level of microRNA expression was determined by qRT-PCR method. Firstly, the reverse transcription reaction was performed in a total volume of 10 μL, prepared as follows: 2 μL of isolated total RNA, 2 μL of 5× RT Reaction Buffer, 1 μL of enzyme mix, and 5 μL of RNase-free water using the miRCURY LNA RT Kit (Qiagen, Germantown, MD, USA), following the manufacturer’s guidelines [56]. The reverse transcription reaction was conducted for 60 min on a GeneAmp PCR System 9700 thermal cycler (Applied Biosystems, Foster City, CA, USA) at 40 °C, followed by a 5-min inactivation step at 95 °C. After completion of the reverse transcription, reaction mixture was cooled to 4 °C and either used immediately for qPCR or stored at −20 °C.

Secondly, the expression profiling was carried out with the miRCURY LNA miRNA Probe PCR Assay (Qiagen, USA) on a 7900HT Fast Real-Time PCR System (Applied Biosystems, Foster City, CA, USA). For the subsequent real-time qPCR, each 10 μL reaction contained 5 μL of 2× miRCURY LNA SYBR^®^ Green Master Mix, 1 μL of cDNA template, 0.5 μL of miRNA-specific primer, and 3.5 μL of RNase-free water. The reaction was carried out under the following thermal conditions: an initial step at 95 °C for 2 min, followed by 40 cycles at 95 °C for 5 s and 56 °C for 30 s. Finally, the data were processed using SDS 2.4 and Data Assist v3.01 software (Applied Biosystems, Foster City, CA, USA) and normalized with the 2^−ΔCt^ method. MiR-16-5p and miR-103a-3p were employed as reference genes.

The assay IDs (Applied Biosystems, Foster City, CA, USA) for the studied microRNA were as follows: has-miR-21-5p (ZP00000445, sequence: UAGCUUAUCAGACUGAUGUUGA), has-miR-30b-5p (ZP00000547, sequence: UGUAAACAUCCUACACUCAGCU), has-miR-103a-3p (ZP00000028, sequence: AGCUUCUUUACAGUGCUGCCUUG), and has-miR-16-5p (ZP00000315, sequence: UAGCAGCACGUAAAUAUUGGCG). Expression levels of the normalized miRNAs are presented as median values with interquartile ranges (25th–75th percentile), as the data distribution did not follow a normal distribution. The stable blood expression of has-miR-103a-3p and has-miR-16-5p supported their use as endogenous controls to evaluate miR-21 and miR-30b expressions [57].

### 4.3. Statistical Analysis

Descriptive statistics for anthropometric, biochemical, and molecular variables—including SOD3, CAT, and microRNA expression levels—are presented as medians with interquartile ranges, reflecting the non-normal distribution of the data. Normality was assessed using the Shapiro–Wilk test. Accordingly, between-group comparisons were performed using the nonparametric Mann–Whitney U test. Associations between molecular markers (SOD3, CAT, and microRNAs) and clinical variables were evaluated using Spearman’s rank correlation coefficient. To identify independent predictors of SOD3, CAT, and microRNA expression, a stepwise forward multiple linear regression analysis was conducted. The relationship between CMD risk and the investigated biomarkers was examined using logistic regression models. Variables not meeting normality assumptions were logarithmically transformed prior to regression analysis to improve model fit. Diagnostic performance was assessed via ROC curve analysis for SOD3, CAT, and microRNA expression levels. AUC was calculated, and optimal cut-off points were established based on Youden’s index. Differences in CMD prevalence between groups stratified by these cut-offs were tested using the chi-square test with Yates’ correction for continuity. All statistical analyses were performed using GraphPad Prism v8.0 (GraphPad Software, San Diego, CA, USA), STATISTICA v13.3 (TIBCO Software Inc., Palo Alto, CA, USA), and MedCalc v22 (MedCalc Software Ltd., Ostend, Belgium). A *p*-value < 0.05 was considered statistically significant.

## 5. Conclusions

In conclusion, our study identifies reduced plasma SOD3 levels as significantly linked with CMD in elderly patients, particularly those with T2DM, though its lack of significant reduction in prediabetes limits its early diagnostic utility. Elevated miR-21 expression showed limited value as a standalone marker due to weak correlation with metabolic parameters, while CAT levels and miR-30b expression did not demonstrate diagnostic relevance. Further research with larger, diverse cohorts is necessary to validate these findings and explore the potential interplay between SOD3 and miR-21 in CMD progression.

## Figures and Tables

**Table 1:** Clinical and biochemical characteristics of participants aged 65 and above in the control (n = 38), prediabetes (n = 37), and type 2 diabetes mellitus (T2DM) (n = 51) groups.

Parameters	Control (n = 38)	Prediabetes (n = 37)	T2DM (n = 51)
***Anthropometric***			
F/M	18/20	22/15	34/17
Age [years]	73.00(67.75; 85.00)	76.00(69.5; 85.5)	75.00(70.00; 82.00)
BMI [kg/m^2^]	26.26(23.68; 31.14)	26.50(24.40; 29.25)	27.25(24.93; 30.93)
ST triceps (mm)	**15.60(10.80; 20.20)**	**17.40(11.10; 24.50)**	**21.80(17.80; 27.60**) ^bb.c^
ST abdominal (mm)	**26.00(18.70; 33.55)**	31.00(19.60; 37.90)	**34.00(26.80; 41.40)** ^b^
ST thigh (mm)	**18.40(14.35; 25.20)**	23.40(11.80; 36.20)	**31.20(17.80; 38.80)** ^b^
BIA-BF [%]	**25.00(20.70; 31.10)**	**25.00(20.70; 32.50)** ^c^	**30.60(24.10; 37.80)** ^b.c^
BIA-FFM [%]	**71.00(64.65; 74.65)**	70.20(62.88; 75.28)	**65.90(59.50; 72.00)** ^b^
BIA-TBW [%]	**52.30(48.60; 56.70)**	52.20(46.90; 55.70)	**48.70(44.50; 51.80)** ^b^
***Metabolic***			
HbA1c [%]	**5.50(5.20; 5.60)**	**6.00(5.80; 6.20)** ^aaa^	**6.50(5.80; 7.60)** ^bbb^
FPG [mmol/L]	**5.16(4.67; 5.37)**	**5.37(4.94; 5.90)**	**6.71(5.78; 9.55)** ^bbb. ccc^
HOMA-IR	**1.88(1.15; 2.83)**	2.41(1.38; 3.80)	**2.68(1.86; 4.86)** ^b^
TG/HDL ratio	1.09(0.67; 1.51)	0.99(0.62; 1.38)	1.16(0.75; 1.75)
Creatinine [μmol/L]	87.52(71.90; 110.60)	89.00(74.15; 109.80)	95.60(69.55; 113.50)
Urea [mmol/L]	**5.85(4.50; 7.64)**	7.08(5.31; 9.38)	**7.67(6.02; 9.54) ^b^**
eGFR [mL/min/1.73 m^2^]	70.69(44.40; 90.40)	61.60(44.00; 85.60)	55.20(44.30; 83.40)
LDL [mmol/L]	2.53(1.79; 3.42)	2.15(1.75; 2.85)	2.13(1.48; 3.14)
HDL [mmol/L]	**1.29(1.01; 1.49)**	1.19(0.95; 1.55)	**1.12(0.87; 1.29) ^b^**
TG [mmol/L]	1.32(0.82; 1.72)	1.08(0.86; 1.54)	1.25(0.91; 1.70)
TC [mmol/L]	4.47(3.74; 5.11)	4.04(3.32; 4.70)	3.64(2.95; 5.21)

List of abbreviations: BIA—bioelectrical impedance analysis, BF—body fat, BMI—body mass index, eGFR—estimated glomerular filtration rate, F—females, FFM—free fat mass (muscle mass), FPG—fasting plasma glucose, HbA1c—glycated hemoglobin, HDL—high-density lipoprotein cholesterol, HOMA-IR—homeostasis model assessment for insulin resistance, LDL—low-density lipoprotein cholesterol, M—males, T2DM—type 2 diabetic subjects, TBW—total body water, TC—total cholesterol, TG—triglycerides. Statistically significant results are shown in bold. ^aaa^ *p* < 0.001 Control vs. Prediabetes; ^bbb^ *p* < 0.001, ^bb^ *p* < 0.01,^b^ *p* < 0.05 Control vs. T2DM; ^c^ *p* < 0.05 Prediabetes vs. T2DM; ^ccc^ *p* < 0.001 Prediabetes vs. T2DM.

**Table 2:** Logistic regression estimates (univariate and multivariate) for predictors of carbohydrate metabolism disorders in individuals ≥ 65 years.

Variable	Univariate			Multivariate		
	Wald’s *p*	OR	95% CI	Wald’s *p*	OR	95% CI
**SOD3**	**0.001**	**0.941**	**0.906–0.976**	**0.012**	**0.948**	**0.909–0.988**
CAT	0.636	1.00	0.999–1.002			
miR-21	0.180	0.648	0.344–1.222			
Age	0.407	1.020	0.974–1.067			
Sex (male)	0.091	0.514	0.238–1.112			
BMI	0.295	1.041	0.966–1.122			
WHR	0.699	2.222	0.039–127.410			
**ST triceps**	**0.018**	**1.062**	**1.010–1.116**	0.184	1.052	0.976–1.134
**ST abdominal**	**0.043**	**1.034**	**1.011–1.069**	0.769	1.007	0.958–1.059
ST thigh	0.070	1.026	0.998–1.054			
BIA-BF [%]	0.117	1.036	0.991–1.083			
BIA-FFM [%]	0.088	0.016	0.001–1.856			
BIA-TBW [%]	0.104	0.005	0.001–1.960			
**FPG**	**<0.001**	**2.585**	**1.500–4.456**	**0.017**	**2.710**	**1.197–6.134**
**HOMA-IR**	**0.045**	**1.794**	**1.012–3.179**	0.802	0.903	0.407–2.00
Creatinine	0.683	1.003	0.990–1.016			
eGFR	0.271	0.991	0.975–1.007			
LDL	0.123	0.737	0.500–1.086			
TG/HDL	0.370	1.264	0.758–2.108			

List of abbreviations: OR—odds ratio, CI—confidence interval, BIA—bioelectrical impedance analysis, BF—body fat, BMI—body mass index, CAT—catalase, eGFR—estimated glomerular filtration rate, FFM—free fat mass (muscle mass), FPG—fasting plasma glucose,SOD3—superoxide dismutase 3, ST—skinfold thickness, TBW—total body water, WHR—waist–hip ratio. The results in bold indicate statistically significant associations.

## Data Availability

The data generated and analyzed during this study are available from the corresponding author upon reasonable request.

## Supplementary Materials

The following supporting information can be downloaded at https://www.mdpi.com/article/10.3390/ijms26094127/s1.
