# Supplementary material for: Dysregulated miR-21/SOD3, but Not miR-30b/CAT, Profile in Elderly Patients with Carbohydrate Metabolism Disorders: A Link to Oxidative Stress and Metabolic Dysfunction
Source: Int J Mol Sci. 2025 Apr 26;26(9):4127. doi: 10.3390/ijms26094127 (PMC12071572; doi:10.3390/ijms26094127)
Supplement: Supplementary file 1 [file ijms-26-04127-s001.zip › ijms-3517379-supplementary.pdf]

Table S1. More detailed clinical and laboratory parameters of the Participants form the control group (n=38), prediabetes group (n=37) and T2DM group (n=51) aged 65 and older.

Parameters	Control (n=38)	Prediabetes (n=37)	T2DM (n=51)
Anthropometrics			
F/M	18/20	22/15	34/17
Age [years]	73.00(67.75; 85.00)	76.00(69.5; 85.5)	75.00(70.00; 82.00)
SBP(mm Hg)	130.00(120.00; 140.00)	130.00(123.50; 139.50)	130.00(125.00; 142.00)
DBP(mm Hg)	79.00(70.00; 80.00)	80.00(70.00; 83.50)	80.00(70.00; 80.00)
Body mass [kg]	77.65(62.75; 85.00)	73.80(61.45; 80.00)	71.70(61.00; 87.60)
Height [m]	1.68(1.56; 1.76)	1.63(1.53; 1.72)	1.62(1.52; 1.70)
BMI [kg/m^2]	26.26(23.68; 31.14)	26.50(24.40; 29.25)	27.25(24.93; 30.93)
WC [cm]	102.50(92.50; 110.50)	98.00(94.00; 105.80)	103.00(94.00; 112.00)
HC [cm]	98.00(90.00; 108.00)	98.00(92.50; 103.00)	97.00(94.00; 108.00)
WHR	1.04(0.95; 1.07)	1.00(0.97; 1.05)	1.03(0.99; 1.06)
ST triceps (mm)	**15.60(10.80; 20.20)**	**17.40(11.10; 24.50)**	**21.80(17.80; 27.60)**$^{bb.c}$
ST abdominal (mm)	**26.00(18.70; 33.55)**	31.00(19.60; 37.90)	**34.00(26.80; 41.40)**^{b}
ST thigh (mm)	**18.40(14.35; 25.20)**	23.40(11.80; 36.20)	**31.20(17.80; 38.80)**^{b}
Visceral Fat Rating	11.00(8.75; 12.25)	11.50(9.00; 14.00)	12.50(9.75; 14.00)
BIA – BF [%]	**25.00(20.70; 31.10)**	**25.00(20.70; 32.50)**^{c}	**30.60(24.10; 37.80)**$^{b.c}$
BIA – BF [kg]	18.60(12.90; 23.93)	18.75(14.43; 23.40)	20.70(16.20; 29.30)
BIA – FFM [%]	**71.00(64.65; 74.65)**	70.20(62.88; 75.28)	**65.90(59.50; 72.00)**^{b}
BIA – FFM [kg]	54.00(45.13; 60.30)	49.50(40.70; 57.81)	47.70(38.48; 61.08)
BIA – TBW [%]	**52.30(48.60; 56.70)**	52.20(46.90; 55.70)	**48.70(44.50; 51.80)**^{b}
BIA – TBW [kg]	40.10(32.10; 46.78)	37.15(29.50; 43.20)	35.90(28.30; 43.88)
Metabolic			
HbA1c [%]	**5.50(5.20; 5.60)**	**6.00(5.80; 6.20)**^{aaa}	**6.50(5.80; 7.60)**^{bbb}
FPG [mmol/l]	**5.16(4.67; 5.37)**	**5.37(4.94; 5.90)**	**6.71(5.78; 9.55)**$^{bbb. ccc}$
HOMA-IR	**1.88(1.15; 2.83)**	2.41(1.38; 3.80)	**2.68(1.86; 4.86)**^{b}
TG/ HDL ratio	1.09(0.67; 1.51)	0.99(0.62; 1.38)	1.16(0.75; 1.75)
Creatinine [µmol/L]	87.52(71.90; 110.60)	89.00(74.15; 109.80)	95.60(69.55; 113.50)
Urea [mmol/L]	**5.85(4.50; 7.64)**	7.08(5.31; 9.38)	**7.67(6.02; 9.54)**^{b}
eGFR [ml/min/1.73 m^2]	70.69(44.40; 90.40)	61.60(44.00; 85.60)	55.20(44.30; 83.40)
LDL [mmol/L]	2.53(1.79; 3.42)	2.15(1.75; 2.85)	2.13(1.48; 3.14)
HDL [mmol/L]	**1.29(1.01; 1.49)**	1.19(0.95; 1.55)	**1.12(0.87; 1.29)**^{b}
TG [mmol/L]	1.32(0.82; 1.72)	1.08(0.86; 1.54)	1.25(0.91; 1.70)
TC [mmol/L]	4.47(3.74; 5.11)	4.04(3.32; 4.70)	3.64(2.95; 5.21)

List of abbreviations: F—females, M—males, T2DM—type 2 diabetic subjects, BMI—body mass index, WHR—waist to hip ratio, WC—waist circumference, HC – hip circumference, ST -skinfold thickness , BIA- bioelectrical impedance analysis, BF-body fat, FFM- free fat mass- muscle mass, TBW – total body water, HbA1c—glycated hemoglobin FPG—fasting plasma glucose, HOMA-IR—homeostasis model assessment for insulin resistance, eGFR—estimated glomerular filtration rate, TC—total cholesterol, LDL-C—low-density lipoprotein cholesterol, HDL-C—high-density lipoprotein cholesterol, TG—triglycerides. Statistically significant results are shown in bold. $^{aaa}p< 0.001$ Control *vs.* Prediabetes; $^{bbb}p< 0.001$; $^{b}p< 0.05$ Control *vs.* T2DM; $^{ccc}p< 0.001$ Prediabetes *vs.* T2DM.

Table S2. Medications used by all study participants (n = 126).

Name of the Agent	Control (n = 38)		Prediabetes (n = 37)		T2DM (n = 51)	
	n	**%**	***n***	**%**	***n***	**%**
Metformin (1–3 g/day)	0	0.00	2	5.41	38	74.51
TZD–pioglitazone (15 mg/day)	0	0.00	0	0.00	8	15.69
iSGLT2–empagliflozin (10 mg/day)	0	0.00	0	0.00	11	21.57

Name of the Agent	Control (n = 38)		Prediabetes (n = 37)		T2DM (n = 51)	
	n	*%*	*n*	*%*	*n*	*%*
αGI–acarbose (150–300 mg/day)	0	0.00	0	0.00	9	17.65
Atorvastatin (10–40 mg/day)	7	18.42	17	45.95	33	64.71
Rosuvastatin (10–40 mg/day)	12	31.58	5	13.51	12	23.53
Fenofibrate (267 mg/day)	0	0.00	0	0.00	4	7.84
Diabetes diet treatment only	0	0.00	15	40.54	1	1.96
1 agent	0	0.00	20	54.05	10	19.61
2 agents	0	0.00	2	5.41	19	37.25
3 agents	0	0.00	0	0.00	17	33.33
4 agents	0	0.00	0	0.00	4	7.84

List of abbreviations: Metformin—in various doses from 1 g to 3 g daily; iSGLT2—sodium-glucose cotransporter-2 inhibitor; TZD—thiazolidinedione; αGI—α-glucosidase inhibitor; 1 agent—one drug from the table; 2 agents—two drugs from the table; 3 agents—three drugs from the table; 4 agents—four drugs from the table.

Table S3a. Correlations between the level of SOD3 and miR-21. anthropometric parameters. SBP and DBP in patients from the control group (n=38), CMD (n=88), prediabetes (n=37) and T2DM (n= 51).

	**Control (n=38)**		**CMD (n=88)**		**Prediabetes (n=37)**		**T2DM (n=51)**	
**Parameters**	**Rho**	**P**	**Rho**	**P**	Rho	**P**	**Rho**	**P**
miR-21	-0.152	0.368	**-0.280**	**0.008**	-0.113	0.504	**-0.400**	**0.004**
Age [years]	**-0.498**	**0.001**	**-0.221**	**0.039**	**-0.337**	**0.041**	-0.143	0.315
SBP (mmHg)	-0.082	0.624	**0.221**	**0.039**	0.164	0.333	**0.288**	**0.040**
DBP (mmHg)	-0.135	0.418	**0.228**	**0.032**	0.207	0.219	0.257	0.069
Body mass [kg]	0.052	0.755	0.130	0.226	0.144	0.395	0.151	0.290
Height [m]	**0.334**	**0.040**	-0.045	0.679	0.010	0.953	-0.081	0.572
BMI [kg/m2]	0.123	0.463	0.148	0.168	0.119	0.483	0.184	0.196
WC [cm]	0.013	0.939	0.129	0.230	0.119	0.484	0.149	0.298
HC [cm]	0.247	0.135	0.076	0.480	0.059	0.728	0.162	0.255
WHR	-0.299	0.068	0.065	0.548	0.185	0.273	-0.034	0.815
ST triceps (mm)	-0.063	0.705	0.119	0.271	0.066	0.697	0.183	0.197
ST abdominal (mm)	-0.159	0.340	0.023	0.833	-0.039	0.821	0.097	0.500
ST thigh (mm)	-0.177	0.288	0.128	0.245	0.123	0.475	0.170	0.238
Visceral Fat Rating	-0.297	0.070	0.104	0.343	-0.099	0.565	0.254	0.076
BIA-BF [%]	-0.295	0.072	0.064	0.557	-0.099	0.564	0.254	0.076
BIA-BF [kg]	-0.144	0.389	0.145	0.182	0.052	0.764	0.263	0.065
BIA-FFM [%]	0.262	0.113	-0.099	0.367	0.024	0.887	-0.259	0.070
BIA-FFM [kg]	0.219	0.187	0.081	0.458	0.244	0.151	-0.021	0.884
BIA-TBW [%]	**0.388**	**0.016**	-0.109	0.318	0.078	0.650	**-0.329**	**0.020**
BIA-TBW [kg]	0.256	0.120	0.025	0.819	0.175	0.308	-0.080	0.583

List of abbreviations: T2DM—type 2 diabetic. BMI—body mass index. WHR—waist to hip ratio. WC—waist circumference. HC – hip circumference. ST -skinfold thickness . BIA- bioelectrical impedance analysis. BF-body fat. FFM- free fat mass- muscle mass. TBW – total body water. Rho - Spearman's rank correlation coefficient. Statistically significant results are shown in bold.

Table S3b. Correlations between the level of SOD3 and metabolic and renal parameters in patients from the control group (n=38), CMD (n=88), prediabetes (n=37) and with T2DM (n=51).

	**Control (n=38)**		**CMD (n=88)**		**Prediabetes (n=37)**		**T2DM (n=51)**	
**Parameters**	**Rho**	**P**	**Rho**	**P**	Rho	**P**	**Rho**	**P**
HbA1c [%]	0.148	0.376	-0.029	0.785	0.003	0.988	-0.072	0.614
FPG [mmol/l]	0.185	0.266	-0.104	0.335	0.030	0.866	-0.146	0.308
HOMA-IR	0.122	0.464	0.111	0.303	0.151	0.373	0.068	0.634
TG/ HDL ratio	0.129	0.440	0.166	0.126	0.184	0.275	0.155	0.289
Creatinine [μmol/L]	**-0.455**	**0.004**	-0.069	0.525	0.081	0.635	-0.179	0.214
Urea [mmol/L]	**-0.386**	**0.017**	-0.070	0.518	0.022	0.899	-0.181	0.209
eGFR [ml/min/1.73 m2]	**0.606**	**0.001**	0.147	0.173	0.030	0.862	0.255	0.074
LDL [mmol/L]	0.282	0.086	0.027	0.803	-0.051	0.767	0.084	0.568
HDL [mmol/L]	-0.155	0.353	-0.125	0.248	**-0.326**	**0.049**	0.030	0.834
TG [mmol/L]	0.187	0.262	0.146	0.178	0.025	0.885	0.252	0.077
T-CH [mmol/L]	0.238	0.151	0.076	0.485	-0.131	0.441	0.239	0.094

List of abbreviations: HbA1c—glycated hemoglobin FPG—fasting plasma glucose. HOMA-IR—homeostasis model assessment for insulin resistance. eGFR—estimated glomerular filtration rate. T-CH—total cholesterol. LDL-C—low-density lipoprotein cholesterol. HDL-C—high-density lipoprotein cholesterol. TG—triglycerides. Rho - Spearman's rank correlation coefficient. Statistically significant results are shown in bold.

Table S3c. Correlations between the level of miR-21 expression and anthropometric parameters, SBP and DBP in patients from the control group (n=38), CMD(n=88). prediabetes (n=37) and T2DM (n =51).

	**Control (n=38)**		**CMD (n=88)**		**Prediabetes (n=37)**		**T2DM (n=51)**	
**Parameters**	**Rho**	**P**	**Rho**	**P**	**Rho**	**P**	**Rho**	**P**
Age [years]	**-0.353**	**0.032**	-0.002	0.986	-0.087	0.610	0.046	0.748
SBP (mmHg)	0.073	0.666	-0.107	0.319	-0.117	0.490	-0.082	0.563
DBP (mmHg)	0.144	0.394	-0.027	0.802	-0.046	0.784	0.012	0.933
Body mass [kg]	-0.150	0.375	0.103	0.341	0.228	0.173	0.014	0.921
Height [m]	0.249	0.135	0.095	0.381	**0.326**	**0.048**	-0.092	0.520
BMI [kg/m2]	**-0.393**	**0.016**	0.003	0.978	-0.006	0.969	0.028	0.845
WC [cm]	-0.253	0.129	-0.014	0.896	-0.006	0.969	-0.005	0.968
HC [cm]	-0.205	0.223	0.066	0.538	0.017	0.919	0.098	0.491
WHR	-0.144	0.393	-0.021	0.843	0.087	0.605	-0.057	0.686
ST triceps [mm]	**-0.399**	**0.014**	**-0.276**	**0.009**	-0.205	0.223	**-0.339**	**0.014**
ST abdominal [mm]	**-0.431**	**0.008**	-0.118	0.269	-0.115	0.498	-0.096	0.500
ST thigh [mm]	**-0.484**	**0.002**	**-0.210**	**0.049**	-0.278	0.095	-0.135	0.343
Visceral Fat Rating	**-0.368**	**0.024**	0.118	0.283	0.229	0.177	-0.004	0.975
BIA-BF [%]	**-0.326**	**0.049**	**-0.238**	**0.027**	-0.328	0.051	-0.135	0.348
BIA-BF [kg]	-0.302	0.069	-0.018	0.105	-0.225	0.187	-0.128	0.376
BIA-FFM [%]	**0.342**	**0.038**	**0.257**	**0.017**	**0.398**	**0.016**	0.130	0.367
BIA-FFM [kg]	0.017	0.921	0.206	0.057	**0.339**	**0.043**	0.119	0.409
BIA-TBW [%]	**0.409**	**0.012**	**0.240**	**0.026**	0.298	0.078	0.143	0.324

BIA-TBW [kg]	0.036	0.833	**0.213**	**0.049**	**0.388**	**0.020**	0.110	0.445

List of abbreviations: T2DM—type 2 diabetic. BMI—body mass index. WHR—waist to hip ratio. WC—waist circumference. HC – hip circumference. ST -skinfold thickness . BIA- bioelectrical impedance analysis. BF-body fat. FFM- free fat mass- muscle mass. TBW – total body water. Rho - Spearman's rank correlation coefficient. Statistically significant results are shown in bold.

Table S3d. Correlations between the level of miR-21 expression and metabolic and renal parameters in patients from the control group (n=38), CMD (n=88), prediabetes (n=37) and with T2DM (n=51).

List of abbreviations: HbA1c—glycated hemoglobin FPG—fasting plasma glucose. HOMA-IR—homeostasis model assessment

	**Control (n=38)**		**CMD (n=88)**		**Prediabetes (n=37)**		**T2DM (n=51)**	
**Parameters**	**Rho**	**P**	**Rho**	**P**	**Rho**	**P**	**Rho**	**P**
HbA1c [%]	0.145	0.391	0.079	0.460	0.198	0.240	0.028	0.841
FPG [mmol/l]	0.001	0.998	-0.067	0.534	0.117	0.486	-0.140	0.326
HOMA-IR	0.032	0.846	0.061	0.572	-0.140	0.408	0.150	0.292
TG/ HDL ratio	0.086	0.609	0.007	0.952	0.021	0.712	0.033	0.821
Creatinine [μmol/L]	-0.107	0.526	0.068	0.530	0.015	0.931	0.108	0.455
Urea [mmol/L]	-0.142	0.401	0.059	0.584	-0.027	0.872	0.122	0.399
eGFR [ml/min/1.73 m2]	0.139	0.409	-0.025	0.815	0.023	0.890	-0.049	0.734
LDL [mmol/L]	0.050	0.767	-0.043	0.692	0.154	0.362	-0.173	0.234
HDL [mmol/L]	0.117	0.487	0.034	0.751	-0.048	0.773	0.088	0.542
TG [mmol/L]	0.182	0.280	0.021	0.844	-0.087	0.606	0.122	0.398
T-CH [mmol/L]	0.156	0.353	-0.024	0.821	0.032	0.847	-0.063	0.663

for insulin resistance. eGFR—estimated glomerular filtration rate. T-CH—total cholesterol. LDL-C—low-density lipoprotein cholesterol. HDL-C—high-density lipoprotein cholesterol. TG—triglycerides. Rho - Spearman's rank correlation coefficient. Statistically significant results are shown in bold.

Table S4a. Correlations between the level of CAT and miR-30b anthropometric parameters, SBP and DBP in patients from the control group (n=38), CMD(n=88). prediabetes (n=37) and T2DM (n =51).

	**Control (n=38)**		**CMD (n=88)**		**Prediabetes (n=37)**		**T2DM (n=51)**	
**Parametry**	**Rho**	**P**	**Rho**	**P**	**Rho**	**P**	**Rho**	**P**
miR-30b	0.008	0.963	0.174	0.110	0.225	0.19	0.153	0.28
SBP [mmHg]	**-0.369**	**0.027**	-0.031	0.775	-0.069	0.693	-0.008	0.954
DBP [mmHg]	**-0.444**	**0.007**	-0.004	0.973	-0.037	0.831	0.001	0.999
Body mass [kg]	-0.107	0.535	**-0.229**	**0.034**	**-0.370**	**0.029**	-0.136	0.341
Heigh [cm]	0.182	0.288	-0.139	0.202	-0.296	0.085	-0.040	0.779
BMI [kg/m^2]	-0.327	0.0519	-0.202	0.063	-0.223	0.198	-0.190	0.186
WC [cm]	-0.108	0.532	-0.034	0.753	-0.070	0.688	-0.034	0.811
HC [cm]	-0.275	0.105	-0.154	0.156	-0.197	0.257	-0.121	0.396
WHR	0.278	0.101	0.0001	0.998	0.005	0.979	0.002	0.987
ST triceps [mm]	**-0.419**	**0.011**	0.101	0.357	0.080	0.647	0.133	0.352
ST abdominal [mm]	-0.180	0.293	-0.006	0.960	-0.137	0.432	0.111	0.437
ST thigh [mm]	**-0.358**	**0.032**	-0.093	0.391	-0.115	0.512	-0.077	0.592

Visceral Fat Rating	-0.308	0.067	-0.202	0.068	-0.272	0.119	-0.074	0.608
BIA-BF [%]	**-0.329**	**0.050**	-0.053	0.635	-0.017	0.925	-0.011	0.937
BIA-BF [kg]	-0.263	0.121	-0.094	0.394	-0.180	0.308	-0.039	0.788
BIA-FFM [%]	**0.350**	**0.036**	-0.016	0.884	0.003	0.985	0.012	0.934
BIA-FFM [kg]	0.093	0.591	-0.138	0.209	-0.309	0.075	-0.169	0.242
BIA-TBW [%]	**0.399**	**0.016**	0.004	0.969	0.090	0.611	0.025	0.864
BIA-TBW [kg]	0.108	0.531	-0.146	0.193	-0.331	0.056	-0.161	0.263

List of abbreviations: T2DM—type 2 diabetic. BMI—body mass index. WHR—waist to hip ratio. WC—waist circumference. HC – hip circumference. ST -skinfold thickness . BIA- bioelectrical impedance analysis. BF-body fat. FFM- free fat mass- muscle mass. TBW – total body water. Rho - Spearman's rank correlation coefficient. Statistically significant results are shown in bold.

Table S4b. Correlations between the level of CAT and metabolic and renal parameters in patients from the control group (n=38). CMD (n=88). prediabetes (n=37) and with T2DM (n=51).

	**Control (n=38)**		**CMD (n=88)**		**Prediabetes (n=37)**		**T2DM (n=51)**	
**Parameters**	**Rho**	**P**	**Rho**	**P**	**Rho**	**P**	**Rho**	**P**
HbA1c [%]	0.065	0.708	-0.209	0.053	-0.080	0.648	-0.258	0.068
FPG [mmol/l]	0.111	0.517	-0.135	0.217	-0.259	0.132	-0.082	0.568
HOMA-IR	-0.089	0.604	-0.006	0.956	-0.018	0.919	-0.038	0.791
TG/ HDL ratio	0.050	0.771	-0.072	0.515	0.009	0.961	-0.055	0.707
Creatinine [µmol/L]	**-0.609**	**0.001**	**-0.234**	**0.031**	-0.050	0.777	**-0.352**	**0.012**
Urea [mmol/L]	-0.257	0.131	**-0.254**	**0.019**	-0.064	0.715	**-0.417**	**0.002**
eGFR [ml/min/1.73 m2]	**0.399**	**0.016**	0.211	0.052	0.031	0.858	**0.335**	**0.017**
LDL [mmol/L]	0.272	0.108	-0.033	0.762	-0.131	0.453	0.048	0.741
HDL [mmol/L]	-0.079	0.649	0.016	0.887	-0.132	0.448	0.049	0.733
TG [mmol/L]	0.034	0.843	-0.070	0.525	-0.033	0.849	-0.049	0.736
T-CH [mmol/L]	0.261	0.124	0.021	0.851	-0.099	0.573	0.098	0.499

List of abbreviations: HbA1c—glycated hemoglobin FPG—fasting plasma glucose. HOMA-IR—homeostasis model assessment for insulin resistance. eGFR—estimated glomerular filtration rate. T-CH—total cholesterol. LDL-C—low-density lipoprotein cholesterol. HDL-C—high-density lipoprotein cholesterol. TG—triglycerides. Rho - Spearman's rank correlation coefficient. Statistically significant results are shown in bold.

Table S4c. Correlations between the level of miR-30b expression and anthropometric parameters. SBP and DBP in patients from the control group (n=38). CMD(n=88). prediabetes (n=37) and T2DM (n =51).

	**Control (n=38)**		**CMD (n=88)**		**Prediabetes (n=37)**		**T2DM (n=51)**	
**Parameters**	**Rho**	**P**	**Rho**	**P**	**Rho**	**P**	**Rho**	**P**
Age	-0.305	0.066	-0.090	0.399	-0.094	0.576	-0.088	0.536
SBP [mmHg]	0.083	0.625	-0.097	0.368	-0.234	0.162	-0.002	0.986
DBP [mmHg]	0.076	0.651	-0.101	0.350	-0.147	0.383	-0.034	0.810
Body mass [kg]	-0.001	0.991	0.105	0.329	0.179	0.287	0.056	0.694
Heigh [cm]	0.146	0.385	0.064	0.552	0.190	0.257	-0.046	0.744

BMI [kg/m2]	-0.097	0.566	0.038	0.728	0.013	0.937	0.070	0.627
WC [cm]	-0.038	0.826	-0.002	0.983	-0.030	0.856	0.016	0.912
HC [cm]	0.092	0.585	0.021	0.841	-0.097	0.569	0.109	0.448
WHR	-0.089	0.599	-0.056	0.607	0.015	0.928	-0.111	0.440
ST triceps [mm]	-0.201	0.230	-0.191	0.074	-0.179	0.288	-0.209	0.140
ST abdominal [mm]	-0.118	0.484	-0.059	0.582	-0.110	0.515	-0.014	0.922
ST thigh [mm]	-0.143	0.395	-0.121	0.261	-0.227	0.176	-0.029	0.835
Visceral Fat Rating	-0.018	0.913	0.085	0.443	0.205	0.229	-0.043	0.764
BIA-BF [%]	-0.010	0.950	-0.141	0.194	-0.213	0.212	-0.138	0.337
BIA-BF [kg]	0.011	0.946	0.013	0.901	-0.169	0.324	-0.104	0.470
BIA-FFM [%]	0.037	0.826	0.144	0.185	0.284	0.093	0.123	0.394
BIA-FFM [kg]	0.004	0.979	0.206	0.056	0.205	0.229	0.164	0.255
BIA-TBW [%]	0.008	0.960	0.169	0.117	0.327	0.051	0.194	0.177
BIA-TBW [kg]	<0.001	0.997	0.076	0.495	0.297	0.078	0.191	0.183

List of abbreviations: T2DM—type 2 diabetic. BMI—body mass index. WHR—waist to hip ratio. WC—waist circumference. HC – hip circumference. ST -skinfold thickness . BIA- bioelectrical impedance analysis. BF-body fat. FFM- free fat mass- muscle mass. TBW – total body water. Rho - Spearman's rank correlation coefficient. Statistically significant results are shown in bold.

Table S4d. Correlations between the level of miR-30b expression and metabolic and renal parameters in patients from the control group (n=38). CMD (n=88). prediabetes (n=37) and with T2DM (n=51).

	**Control (n=38)**		**CMD (n=88)**		**Prediabetes (n=37)**		**T2DM (n=51)**	
**Parameters**	**Rho**	**P**	**Rho**	**P**	**Rho**	**P**	**Rho**	**P**
HbA1c [%]	0.166	0.325	0.024	0.824	-0.076	0.652	0.002	0.987
FPG [mmol/l]	-0.218	0.193	-0.099	0.361	-0.001	0.999	-0.220	0.120
HOMA-IR	<0.001	0.995	0.058	0.590	-0.220	0.190	0.216	0.126
TG/ HDL ratio	0.129	0.445	-0.003	0.977	0.053	0.754	-0.055	0.707
Creatinine [µmol/L]	-0.148	0.379	-0.063	0.559	-0.025	0.881	-0.085	0.552
Urea [mmol/L]	-0.087	0.605	-0.056	0.604	-0.088	0.602	-0.024	0.867
eGFR [ml/min/1.73 m2]	0.243	0.145	0.049	0.650	0.037	0.827	0.062	0.668
LDL [mmol/L]	0.032	0.850	0.005	0.961	0.155	0.359	-0.111	0.445
HDL [mmol/L]	0.108	0.522	0.062	0.570	-0.010	0.951	0.102	0.480
TG [mmol/L]	0.247	0.139	0.037	0.732	0.069	0.682	0.025	0.863

T-CH [mmol/L]	0.184	0.274	-0.004	0.969	0.087	0.607	-0.071	0.625

List of abbreviations: HbA1c—glycated hemoglobin FPG—fasting plasma glucose. HOMA-IR—homeostasis model assessment for insulin resistance. eGFR—estimated glomerular filtration rate. T-CH—total cholesterol. LDL-C—low-density lipoprotein cholesterol. HDL-C—high-density lipoprotein cholesterol. TG—triglycerides. Rho - Spearman's rank correlation coefficient. Statistically significant results are shown in bold.
